# Development of Prediction Models for Antenatal Care Attendance in Amhara Region, Ethiopia

**DOI:** 10.1001/jamanetworkopen.2023.15985

**Published:** 2023-05-31

**Authors:** Bryan Wilder, Clara Pons-Duran, Frederick G. B. Goddard, Bezawit Mesfin Hunegnaw, Sebastien Haneuse, Delayehu Bekele, Grace J. Chan

**Affiliations:** 1 Department of Epidemiology, Harvard T. H. Chan School of Public Health, Boston, Massachusetts; 2 Machine Learning Department, Carnegie Mellon University, Pittsburgh, Pennsylvania; 3 Department of Pediatrics and Child Health, St Paul’s Hospital Millennium Medical College, Addis Ababa, Ethiopia; 4 Department of Biostatistics, Harvard T. H. Chan School of Public Health, Boston, Massachusetts; 5 Department of Obstetrics and Gynecology, St Paul’s Hospital Millennium Medical College, Addis Ababa, Ethiopia; 6 Division of Medicine Critical Care, Boston Children’s Hospital, Department of Pediatrics, Harvard Medical School, Boston, Massachusetts

## Abstract

**Importance:**

Antenatal care prevents maternal and neonatal deaths and improves birth outcomes. There is a lack of predictive models to identify pregnant women who are at high risk of failing to attend antenatal care in low-resource settings.

**Objective:**

To develop a series of predictive models to identify women who are at high risk of failing to attend antenatal care in a rural setting in Ethiopia.

**Design, Setting, and Participants:**

This prognostic study used data from the Birhan Health and Demographic Surveillance System and its associated pregnancy and child cohort. The study was conducted at the Birhan field site, North Shewa zone, Ethiopia, a platform for community- and facility-based research and training, with a focus on maternal and child health. Participants included women enrolled during pregnancy in the pregnancy and child cohort between December 2018 and March 2020, who were followed-up in home and facility visits. Data were analyzed from April to December 2022.

**Exposures:**

A wide range of sociodemographic, economic, medical, environmental, and pregnancy-related factors were considered as potential predictors. The selection of potential predictors was guided by literature review and expert knowledge.

**Main Outcomes and Measures:**

The outcome of interest was failing to attend at least 1 antenatal care visit during pregnancy. Prediction models were developed using logistic regression with regularization via the least absolute shrinkage and selection operator and ensemble decision trees and assessed using the area under the receiving operator characteristic curve (AUC).

**Results:**

The study sample included 2195 participants (mean [SD] age, 26.8 [6.1] years; mean [SD] gestational age at enrolment, 25.5 [8.8] weeks). A total of 582 women (26.5%) failed to attend antenatal care during cohort follow-up. The AUC was 0.61 (95% CI, 0.58-0.64) for the regularized logistic regression model at conception, with higher values for models predicting at weeks 13 (AUC, 0.68; 95% CI, 0.66-0.71) and 24 (AUC, 0.66; 95% CI, 0.64-0.69). AUC values were similar with slightly higher performance for the ensembles of decision trees (conception: AUC, 0.62; 95% CI, 0.59-0.65; 13 weeks: AUC, 0.70; 95% CI, 0.67-0.72; 24 weeks: AUC, 0.67; 95% CI, 0.64-0.69).

**Conclusions and Relevance:**

This prognostic study presents a series of prediction models for antenatal care attendance with modest performance. The developed models may be useful to identify women at high risk of missing their antenatal care visits to target interventions to improve attendance rates. This study opens the possibility to develop and validate easy-to-use tools to project health-related behaviors in settings with scarce resources.

## Key Points

**Question:**

Is it possible to accurately predict risk of failure to attend antenatal care in Amhara region, Ethiopia?

**Findings:**

In this prognostic study that used data from 2195 pregnant women, the predictive performance of the 6 predictive models fit was modest, with the areas under the receiving operator characteristic curves ranging from 0.61 to 0.70.

**Meaning:**

These findings suggest that it may be possible to develop predictive models to identify women at high risk of failing to attend antenatal care to target specific interventions aiming to improve antenatal care attendance rates, using data from surveillance systems and pregnancy cohorts in low-resource settings.

## Teaser

This prognostic study develops and describes a series of predictive models to identify women who are at high risk of failing to attend antenatal care in a rural setting in Ethiopia.

## Introduction

Antenatal care is the provision of essential maternity services and interventions aiming to prepare the mother for the birth, to ensure the healthy development of the pregnancy, and to prevent, diagnose, and anticipate complications.^1^ Evidence shows that antenatal care provision has benefits in preventing maternal and neonatal deaths^2,3,4^ and improving birth outcomes.^5,6^ Since the 1990s, antenatal care attendance has been based on a model of a minimum of 4 visits.^1^ Based on evidence suggesting that additional visits decrease perinatal mortality and increase mother’s satisfaction,^7^ the World Health Organization (WHO) launched its most recent antenatal care guidelines in 2016, recommending 8 contacts during pregnancy. Despite the new WHO model, country and regional estimates regarding antenatal care attendance are only available for the focused antenatal care model in most countries. In Ethiopia, the latest estimates from 2019 Ethiopia Mini Demographic and Health Survey (EMDHS) indicate that 74% of women aged 15 to 49 years who had a live birth in the 5 years before the survey had attended at least 1 skilled antenatal care visit. However, the percentage of women who had at least 4 antenatal care visits was lower, at 43%.^8^ The proportions varied significantly across country regions and between urban and rural areas. Urban women were more likely to attend at least 4 antenatal care visits (59%) than women living in rural areas (37%). Of note, the EMDHS estimates were all based on self-reported information.

There is a large body of evidence about the factors associated with antenatal care attendance and failure. Available studies found that socioeconomic status, educational level, distance from the health center, and marital status, among others, were associated with access to antenatal care services in Ethiopia.^9,10^ Additionally, obstetric and medical history–related factors, like previous history of stillbirth, were associated with a reduced attendance rate to antenatal care services in subsequent pregnancies.^11^ The first step toward designing and implementing strategies to encourage women to attend antenatal care services is to identify individuals who may be more prone to skip visits based on their background characteristics, health history, and status. To our knowledge, no previous studies have developed predictive models that address this need.

It is critical to ensure universal access to quality antenatal care services and to identify population groups that are less likely to access those services in settings with low antenatal care coverage rates. Stratifying women at risk for low antenatal care coverage could target interventions to improve antenatal care attendance and to mobilize resources for populations at highest risk. In this context, we conducted a prognostic study using a pregnancy and birth cohort with health and demographic surveillance in the Amhara region of Ethiopia. Our study aims to develop a series of predictive models to identify women who are at high risk of failing to attend antenatal care in rural Amhara, Ethiopia, using information on factors collected at different time points before and during pregnancy at community and facility levels.

## Methods

This prognostic study was approved by the ethics review board of Saint Paul’s Hospital Millennium Medical College (Addis Ababa, Ethiopia), and Harvard T.H. Chan School of Public Health (Boston, Massachusetts). Signed informed consent was obtained from all participants. We followed the Transparent Reporting of a Multivariable Prediction Model for Individual Prognosis or Diagnosis (TRIPOD) reporting guideline.

### Study Design and Setting

This cohort study was conducted in the Birhan field site, a study area that covers 16 villages in the Amhara region of Ethiopia, with a population of 77 766. The site is a platform for community- and facility-based research and training that was established in 2018, with a focus on maternal and child health. The catchment area is rural and semiurban, covers both highland and lowland areas, and includes 2 different districts, Angolela Tera and Kewet/Shewa Robit.^12^

Within the field site, the Birhan Health and Demographic Surveillance System (Birhan HDSS) conducts house-to-house surveillance every 3 months on individual demographic and health information, including pregnancy status.^12^ Nested in the HDSS is an open pregnancy and birth cohort (Birhan Cohort) that enrolls approximately 2000 pregnant women and their newborns every year, with scheduled home visits and passive facility follow-up. Children are rigorously followed-up over the first 2 years of life.^13^ We used data from the Birhan HDSS and Cohort to develop a series of prediction models for antenatal care attendance. Detailed information about the study design and procedures of the Birhan HDSS and Cohort have been published elsewhere.^12,13^

### Study Population

We used data of women enrolled during pregnancy in the Birhan Cohort between December 2018 and March 2020. Of those, we excluded women who delivered after April 9, 2020, since antenatal care visits were no longer recorded in the facility records after the start of COVID-19 pandemic restrictions in the study area. We excluded newborns with implausible gestational ages at birth, ie, less than 28 weeks due to the definition of stillbirth in Ethiopia and 46 weeks or longer. Gestational age was estimated following a specific hierarchy. If an ultrasonographic measurement at less than 16 weeks’ gestation was available, that measurement was used to estimate gestational age. If ultrasonographic data were only available after 16 weeks’ gestation, self-reported last menstrual period (LMP) was used if there was a discrepancy between the LMP and ultrasonographic estimates. If no ultrasonographic estimates were available, LMP was used, and if neither of those methods were available, then fundal height was used over a maternal estimate (reported in months).^14^

### Outcomes

The principal outcome was failing to attend at least 1 antenatal care visit during pregnancy. In the cohort, antenatal care visits that took place in the study catchment area during pregnancy follow-up after enrollment were captured by prospective facility record abstraction. In a subset of participants, data collectors abstracted antenatal care visits from facility records retrospectively to minimize missing data from visits that occurred prior to enrollment and were missed by data collectors. Visits with a record in the mothers’ medical record, abstracted either retrospectively or prospectively, were used to create the outcome variable of the predictive models. Of note, some antenatal care visits before enrollment and those that happened outside of the study catchment area may have been missed from facility record abstraction.

### Potential Predictors

The initial selection of potential predictors was guided by a literature review of papers published between 1990 and 2022 reporting on factors associated with antenatal care attendance in sub-Saharan Africa. We searched PubMed using the following search terms: (*prenatal* OR *antenatal* OR *ANC*) AND (*risk factors* OR *determinants*). Additional factors collected as part of the Birhan HDSS and Cohort were also considered for inclusion. Following the removal of potential predictors with ambiguous definitions and low prevalence rates in the data (ie, <5 cases), a range of socioeconomic, demographic, medical, environmental, and pregnancy-related potential predictors were considered for inclusion in the predictive models. The complete list of included potential predictors can be found in eTable 1 in Supplement 1.

### Descriptive Analysis

A descriptive analysis of the background characteristics of study participants was performed. Binary variables were reported using counts and percentages; continuous variables were reported using median and interquartile range (IQR).

### Statistical Analysis

#### Prediction Models

Supervised classification models were fit to predict the binary outcome of whether a woman will have at least 1 antenatal care visit during pregnancy. The positive outcome denotes that a woman had no antenatal care visits, as the goal was to identify women who are at risk of receiving no antenatal care. Two kinds of models were fit: a logistic regression model with regularization via the least absolute shrinkage and selection operator (LASSO) to avoid overfitting^15^; an ensemble of decision trees (XGBoost), which can automatically leverage nonlinear interactions between potential predictors.^16^ All models were fit and internally validated using 5-fold cross validation.^17^ Both models perform a form of variable selection during fitting, the LASSO via penalizing model coefficients toward zero and decision trees by selecting a small subset of variables to branch on. To report CIs for the logistic model, we subsequently fit an unregularized logistic regression model to the variables selected via LASSO (eTable 2 in Supplement 1), as regularization invalidates standard CIs.^18^ Odds ratios (OR) and 95% CIs were only reported for variables with at least 10 observations in each level, since unregularized point estimates and 95% CIs are highly unstable for potential predictors with very few observations in some levels.

The discrimination of all models was estimated using the area under the receiving operator characteristic (ROC) curve (AUC) on the held-out data within each cross-validation fold. An AUC value of 0.5 represents a random prediction that is uncorrelated with the true outcome. Larger values indicate more accurate predictions, and a value of 1 represents predictions that are perfectly concordant with the true outcome. ROC curves were generated to assess the potential clinical utility of the models by measuring its specificity at a fixed level of sensitivity.

We compared the predictive performance that can be achieved by each model as a function of the gestational age at which a prediction is made. This measures the clinical utility of the model in a scenario where risk prediction is performed at a given point in pregnancy, using all information available up until that point. At a given time *t*, we predicted attendance only for women who did not attend any antenatal care visits by *t*. This assesses the ability of the model to differentiate between women for whom it is still unknown (at *t*) whether they will receive antenatal care. We considered 3 distinct time points: *t* = 0, ie, using only information available at the time of conception, *t* = 13 weeks, and *t* = 24 weeks.

#### Missing Data Handling

Ensemble of decision trees automatically handle missing values for the potential predictors by picking a default branch in each tree along which to route observations with missing values. For logistic regression with LASSO, we performed multiple imputation via the Multivariate Imputation by Chained Equations (MICE) package for R.^19^ MICE iteratively estimates the conditional distribution of each variable given the others using a regression model and imputes missing values with predictions from this model. It provides unbiased estimates when data satisfies a missing at random assumption; however, as our goal is prediction, biased estimates of missing values may still improve predictive performance even if this assumption is not met. Before performing imputation, dummy variables indicating missingness for each potential predictor were included as additional variables, an approach that is justified for predictive models because it reflects the complete state of knowledge available at the time of prediction.

Analyses were conducted using R statistical software version 4.1.2 (R Project for Statistical Computing). Data were analyzed from April to December 2022.

## Results

The initial sample was composed of 2801 women. We first removed 73 individuals (2.6%) with extreme gestational ages and 2 individuals (0.1%) who did not have information on gestational age. Then, 475 women of the remaining 2726 (17.4%) delivered after the onset of COVID-19 pandemic, or were expected to deliver after that date in case of censored participants, and were also excluded from the study. Of the remaining 2251 women, we excluded 56 women (2.5%) who were lost to follow-up before delivery and before attending any antenatal care visits. The final study sample size was 2195 participants (mean [SD] age, 26.8 [6.1] years) (Table 1). Almost half of the sample were women who could not read and write (983 women [44.9%]). There was an even representation of women from both districts in the study area (951 women [43.4%] from Angolela Tera district), and almost one-third of participants were in their first pregnancy (709 women [32.3%] were primiparous). A total of 582 study participants (26.5%) did not have any recorded antenatal care visits during pregnancy (prospective or retrospective).

**Table 1.** Characteristics of the Study Sample

Variables	Participants, No. (%) (N = 2195)
Age at conception, mean (SD), y^a^	26.8 (6.1)
Gestational age at enrolment, mean (SD), wk	25.9 (8.8)
Cannot read and write^b^	983 (44.9)
District: Angolela Tera^c^	951 (43.4)
Primiparous	709 (32.3)
No recorded antenatal care visits	582 (26.5)

^a^ Includes data for 2189 participants.

^b^ Includes data for 2190 participants.

^c^ Includes data for 2191 participants.

Some potential predictors had high levels of missing data, especially when we relied on information collected by the health system (eg, signs and symptoms) for the week 13 and 24 models. Not all included individuals were enrolled by weeks 13 and 24. Approximately 90% of participants missed data on signs and symptoms for the week 13 model, and approximately 60% of participants had missing data on signs and symptoms for the week 24 model. Anthropometric measurements collected before pregnancy were missing for more than 55% of the cohort because those were not collected in all HDSS rounds. Approximately 10% of women had missing data on nutritional habits.

Models were fit to predict the probability that each woman would attend no antenatal care visits, varying the time (measured in gestational age) at which the prediction was made. The Figure shows the ROC curves for the logistic regression with LASSO and the ensemble of decision trees at each time point. The AUC was 0.61 (95% CI, 0.58-0.64) for the model at conception, with higher values for models predicting at weeks 13 (AUC, 0.68; 95% CI, 0.66-0.71) and 24 (AUC, 0.66; 95% CI, 0.64-0.69) (Table 2). ROC curves and AUC values were generally similar between the 2 models, with slightly higher performance for the ensemble of decision trees (conception: AUC, 0.62; 95% CI, 0.59-0.65; 13 weeks: AUC, 0.70; 95% CI, 0.67-0.72; 24 weeks: AUC, 0.67; 95% CI, 0.64-0.69), although any differences in AUC values were contained within the 95% CI.

**Figure.**
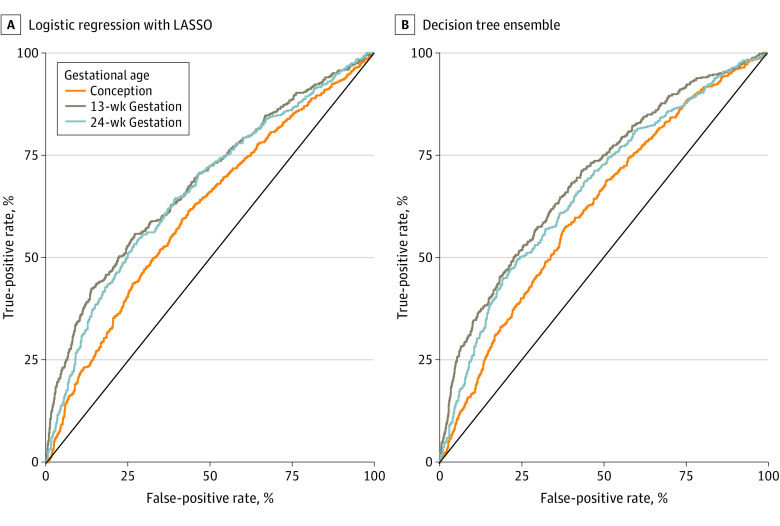
Receiver Operator Characteristic Curves for Each Predictive Model and Time of Prediction LASSO indicates least absolute shrinkage and selection operator.

**Table 2.** AUC of Predictive Models

Time	AUC (95% CI)	
	Logistic regression with LASSO	Ensemble of decision trees
Conception	0.61 (0.58-0.64)	0.62 (0.59-0.65)
Week 13	0.68 (0.66-0.71)	0.70 (0.67-0.72)
Week 24	0.66 (0.64-0.69)	0.67 (0.64-0.69)

Abbreviations: AUC, area under the receiving operator characteristic curve; LASSO, least absolute shrinkage and selection operator.

Table 3 shows the OR of the logistic regression with LASSO at each time point, and Table 4 shows the feature importance for the tree ensembles for the fifteen potential predictors with the strongest associations with the outcome. We observed that demographic features (eg, age, religion), dietary habits, income sources, use of contraception prior to pregnancy, anthropometrics, missing information on obstetric history, and distance to health facilities all consistently played a large role in the predictive models. At later time points (13 and 24 gestational weeks), not receiving community health visits from study data collectors were associated with lack of facility antenatal care attendance (week 13: OR, 5.70; week 24: OR, 6.69). ORs and their 95% CIs for the unregularized regression after variable selection via LASSO can be found in eTable 2 in Supplement 1.

**Table 3.** Odds Ratios for Top Predictors From Logistic Regression With Least Absolute Shrinkage and Selection Operator

Variable	Odds ratio		
	Conception	Week 13	Week 24
Prior contraceptive use	0.67	0.77	0.87
Eat fortified food weekly	0.77	0.70	0.87
No education	1.16	1.22	1.14
Missing time to health facility	4.14	1.17	NA
Income source: merchant	1.41	1.29	NA
Income source: private business	2.09	1.60	NA
Missing interpregnancy interval	0.84	0.65	NA
No community visit	NA	4.05	4.05
Occupation: petty trade	0.83	0.89	0.59
Occupation: self-employed	1.42	1.51	NA
History of baby with birth defects	4.32	1.80	NA
Previously living in a different region (migrated)	0.40	NA	NA
Urinary urgency	NA	2.39	NA

Abbreviation: NA, not applicable.

**Table 4.** Feature Importance for Top Predictors in the Ensemble of Decision Tree

Variable	Importance function^a^		
	Conception	Week 13	Week 24
Age at conception	0.05	0.05	0.04
Body mass index	0.03	0.05	0.03
Prior contraceptive use	0.06	0.03	0.02
Time living at current residence	0.04	0.02	0.04
Higher education	0.01	0.04	0
Family size	0.02	0	0.03
Height	0.05	0.03	0.03
Distance to health facility	0.04	0.03	0.01
Time to health facility	0.18	0.1	0.07
Mid–upper arm circumference	0	0.03	0.01
Muslim	0.04	0.02	0.01
No community visit	NA	0.25	0.33
Age of first pregnancy	0.01	0.01	0.03
Weight	0.08	0.04	0.01

Abbreviation: NA, not applicable.

^a^ Importance is measured via the gain in the XGBoost objective function from splitting on a given feature, with a mean calculated over all trees.

## Discussion

This prognostic study found that, despite the low specificity of the models, it was possible to develop predictive models for behavioral outcomes, such as antenatal care attendance, using data from HDSS and pregnancy cohorts in a setting where there is usually lack of information on key pregnancy-related health indicators and a complete reliance on self-reports to assess health services utilization. The developed models predicted with modest performance the probability that a woman from the Birhan field site did not initiate antenatal care during pregnancy at 3 prediction times. The best performing algorithm was an ensemble of decision trees that obtained an AUC of 0.70 for the prediction at week 13 of gestation. This AUC can be interpreted as the probability that a randomly selected woman who will never attend an antenatal care visit is correctly classified as presenting higher risk to fail to attend antenatal care than a randomly selected woman who will access the services. With the available data, prediction at other time points is poorer.

To our knowledge, this is the first study aiming to develop an accurate model to predict failure to access antenatal care services in a low-resource setting. Previous studies carried out in Ethiopia and other low-resource areas focused on assessing coverage and factors associated with antenatal care attendance but did not report on performance of models or internally validation of such models.^9,10^ In an effort to build the most accurate and well-performing models possible with available data, we used socioeconomic, demographic, obstetric, and medical history–related factors in our study, as well as habits and nutritional factors. Factors of all these different domains were found among the top predictors of failure to attend antenatal care in our models. For instance, women who reported use of contraceptives before getting pregnant or eating fortified foods were less likely to fail to attend antenatal care visits. Participants who did not know how far they lived from the nearest health facility or who got their main income from work in a private company were more prone to not initiate their antenatal care follow-up. With regard to obstetric history, the most important predictor of failure to use antenatal care was having had a baby with birth defects. These findings are in line with previous evidence showing that antenatal care attendance is generally associated with increased socioeconomic level, planned pregnancies and use of contraceptive methods, and no history of complications, such as stillbirth.^4,9,10,11,20,21^ Causes of failure to attend antenatal care need to be further studied beyond association studies. Prediction studies like this aim to characterize prognosis, and a different study design and analytical approach is required to infer causality.

Despite the wide range of potential predictors considered, the models still had modest results that could benefit from additional data. An increase in sample size may contribute to keeping potential predictors that were excluded from the study due to low prevalence rates and thus, potentially improve the model’s predictive performance. We hypothesize that it may be necessary to use data on trust in the health system, such as health services utilization before pregnancy, and health care–seeking behavior, like antenatal care attendance in previous pregnancies, to enhance the predictive ability of the algorithms. Nevertheless, irrespective of the modest performance of the models and the need for additional data to improve it, our study stands as relevant due to its longitudinal design and the novelty in developing the first predictive models for antenatal care attendance in this setting, to our knowledge.

The development of prediction models for antenatal care attendance may have implications for future research and operational activities in countries with strong community health programs, such as Ethiopia. Our data suggest that not receiving pregnancy follow-up visits in the community may predict propensity to not initiate facility antenatal care visits. The Ethiopian government has been implementing the Health Extension Program since 2003, in which health extension workers and the Health Development Army have a central role in health promotion at primary level.^22^ Globally, the most common task carried out by community health workers (CHWs) is health promotion,^23^ and the availability of easy-to-handle tools that allow them to identify women at high risk of not attending antenatal care and disregarding recommendations, may be of high impact. Although one of their responsibilities is already to encourage women to attend antenatal care facilities, experiences from other sub-Saharan African countries suggest that CHWs programs do not always translate into increased utilization rates.^24,25^ Voluntarism, high workload, and burnout of certain profiles of CHWs in Ethiopia are barriers to expand their scope of work and provide additional counselling to all women at community level.^26,27^

Prediction models with modest performance may be used to identify pregnant individuals at a high risk of never initiating antenatal care, and thus those individuals could be targeted in specific interventions to improve antenatal care attendance rates. However, the lack of accuracy of our models may imply that a considerable proportion of individuals at moderate to high risk still will not be targeted in such interventions, but the strategy of intensifying efforts toward a subset of the population might be feasible and cost-effective. In light of our results that show a very similar performance across the models, the ease of interpretation of these models should be taken into account when selecting the best algorithm to be implemented in the field. ORs of logistic regressions may be more interpretable than decision-tree models by the end users.

### Limitations

This study has some limitations. The counts used to generate antenatal care coverage may underestimate the total number of visits attended due to possible visits outside the study catchment area or incomplete retrospective data abstraction. This potential underestimation of antenatal care visits may affect the results of the prediction model by conservatively predicting that women did not receive antenatal care when their visits were simply unlikely to be recorded. Nevertheless, we suspect it is uncommon for women to seek care at facilities outside their residential area. The need to exclude pregnancies that ended after the onset of COVID-19 pandemic must be acknowledged, since it reduced the study sample size. However, we assumed our results may be applicable to the current situation in the Birhan field site, where pregnant women may behave similarly as during the prepandemic years. Furthermore, the level of missing data in some of the potential predictors may be considered a caveat of the study. However, missing data were handled using statistical procedures (eg, MICE for LASSO models). Although the study goal was not to provide inferential guarantees, the 95% CIs of unregularized logistic regressions following variable selection with LASSO need to be interpreted with caution because they do not consider the challenge of postselection inference.^18^ Lack of external validation is a weakness of many published studies on predictive model development. To our knowledge, there are no existing data sets that could be used to externally validate our models. Our models were internally validated using cross-validation to reduce the probability of model overfitting due to the use of a single data set. Future studies are needed to externally validate these models in other populations.

## Conclusion

Our prognostic study presents a series of prediction models for antenatal care attendance that had modest performance. Due to their low specificity, these models are most appropriate for the identification of women at particularly high risk of not using antenatal care services and are less able to differentiate between women at moderate risk. Our study opens the possibility to start exploring the development and validation of easy-to-use tools to predict health-related behaviors in settings with scarcity of resources.

## Notes

Supplement 1. **eTable 1. **Complete List of Predictors Included in the Models **eTable 2.** Unregularized Logistic Regression Model Fit to the Variables Selected Via LASSO: Odds Ratios and 95% CIs for Top Predictors

Supplement 2.
Data Sharing Statement
