# Supplementary material for: Development of Prediction Models for Antenatal Care Attendance in Amhara Region, Ethiopia
Source: JAMA Netw Open. 2023 May 31;6(5):e2315985. doi: 10.1001/jamanetworkopen.2023.15985 (PMC10233415; doi:10.1001/jamanetworkopen.2023.15985)
Supplement: Supplement 1. — eTable 1. Complete List of Predictors Included in the Models eTable 2. Unregularized Logistic Regression Model Fit to the Variables Selected Via LASSO: Odds Ratios and 95% CIs for Top Predictors [file jamanetwopen-e2315985-s001.pdf]

Supplemental Online Content

Wilder B, Pons-Duran C, Goddard FGB, et al. Development of prediction models for antenatal care attendance in Amhara region, Ethiopia. *JAMA Netw Open*. 2023;6(5):e2315985. doi:10.1001/jamanetworkopen.2023.15985

eTable 1. Complete List of Predictors Included in the Models

eTable 2. Unregularized Logistic Regression Model Fit to the Variables Selected Via LASSO: Odds Ratios and 95% CIs for Top Predictors

This supplemental material has been provided by the authors to give readers additional information about their work.

© 2023 Wilder B et al. *JAMA Network Open.*

eTable 1. Complete List of Predictors Included in the Models

Predictors	Models by time point of prediction		
	Conception	Week 13	Week 24
**Socioeconomic and demographic**			
Maternal age	X	X	X
Maternal education	X	X	X
Maternal literacy	X	X	X
Marital status	X	X	X
Occupation	X	X	X
Family's main source of income	X	X	X
Family wealth index	X	X	X
Family size (number of individuals in the household)	X	X	X
Ethnicity	X	X	X
Religion	X	X	X
Woreda	X	X	X
Time and distance to the nearest health facility	X	X	X
Previous residence	X	X	X
Time she has been living in study area	X	X	X
**Anthropometric measurements**			
Preconception weight	X	X	X
Height	X	X	X
Preconception body mass index	X	X	X
Preconception mid-upper arm circumference	X	X	X

© 2023 Wilder B et al. *JAMA Network Open.*

**Environmental and behavioral**			
Individual frequency of tv and radio use	X	X	X
Individual ownership of mobile phone	X	X	X
Alcohol consumption	X	X	X
Khat consumption (stimulant drug)	X	X	X
Nutritional habits (frequency of intake of several products)	X	X	X
Availability of food to feed children in last 30 days	X	X	X
Availability of food to feed herself in last 30 days	X	X	X
Use of contraceptives before pregnancy	X	X	X
**Medical and obstetric history**			
Past medical history of diabetes	X	X	X
Past medical history of sexually transmitted diseases	X	X	X
History of stillbirth	X	X	X
History of miscarriage	X	X	X
History of preterm birth	X	X	X
History of multiple gestation	X	X	X
History of cesarean section	X	X	X
History of low birth weight newborn	X	X	X
History of baby with birth defects	X	X	X
Gravidity	X	X	X

© 2023 Wilder B et al. *JAMA Network Open.*

Parity	X	X	X
Interpregnancy interval	X	X	X
Location of last delivery	X	X	X
Age at first pregnancy	X	X	X
Last pregnancy was planned	X	X	X
Experience of past infant deaths	X	X	X
Last child born is still alive	X	X	X
**Signs and symptoms during pregnancy**			
Vomiting and severe nausea		X	X
Headache		X	X
Blurry vision		X	X
Right upper quadrant abdominal pain		X	X
Urinary pain		X	X
Increased urinary frequency		X	X
Urinary urgency		X	X
Decreased fetal movement		X	X
Vaginal bleeding		X	X
Iron and folic acid supplementation		X	X
**Operations-related**			
Community visits by study field workers during pregnancy		X	X

© 2023 Wilder B et al. *JAMA Network Open.*

eTable 2. Unregularized Logistic Regression Model Fit to the Variables Selected Via LASSO: Odds Ratios and 95% CIs for Top Predictors

**Variable**	**Conception**<br>*OR (95%CI)*	**Week 13**<br>*OR (95%CI)*	**Week 24**<br>*OR (95%CI)*
Prior contraceptive use	0.62 (0.48 - 0.80)	0.64 (0.49 - 0.84)	0.73 (0.55 - 0.98)
Eat cereals weekly	0.54 (0.38 - 0.78)	0.81 (0.56 - 1.18)	-
Eat dairy weekly	1.78 (1.15 - 2.76)	-	-
Eat eggs weekly	1.44 (0.86 - 2.40)	1.61 (0.93 - 2.74)	-
Eat fortified food weekly	0.72 (0.54 - 0.96)	0.64 (0.48 - 0.85)	0.73 (0.53 - 1.01)
Education = None	1.28 (0.96 - 1.71)	1.42 (1.08 - 1.87)	1.35 (1.01 - 1.81)
Income source = Merchant	2.62 (1.35 - 5.00)	2.08 (1.12 - 3.83)	-
Income source = Private	2.73 (1.17 - 6.30)	2.86 (1.24 - 6.61)	-
Interpregnancy interval = missing	0.76 (0.53 - 1.08)	0.50 (0.33 - 0.73)	-
Khat usage	0.31 (0.09 - 0.91)	-	-
Location of last delivery = home	1.36 (1.02 - 1.82)	1.43 (1.08 - 1.89)	1.16 (0.79 - 1.70)
No community visit	-	5.70 (4.09 - 8.00)	6.69 (4.65 - 9.76)
Ethnicity	2.08 (1.27 - 3.38)	1.79 (1.05 - 3.01)	1.52 (0.85 - 2.73)
Occupation = Petty Trade	0.46 (0.06 - 1.76)	0.33 (0.04 - 1.36)	-
Previously leaving in a different region (migrated)	0.19 (0.02 - 0.95)	-	-

CI – confidence interval; OR – odds ratio

© 2023 Wilder B et al. *JAMA Network Open.*
