# Supplementary material for: Development of Prediction Models for Antenatal Care Attendance in Amhara Region, Ethiopia
Source: JAMA Netw Open. 2023 May 31;6(5):e2315985. doi: 10.1001/jamanetworkopen.2023.15985 (PMC10233415; doi:10.1001/jamanetworkopen.2023.15985)
Supplement: Supplement 2. — Data Sharing Statement [file jamanetwopen-e2315985-s002.pdf]

Data Sharing Statement

Wilder. Development of Prediction Models for Antenatal Care Attendance in Amhara Region, Ethiopia. *JAMA Netw Open*. Published May 31, 2023.
doi:10.1001/jamanetworkopen.2023.15985

Data
Data available: No

Additional Information
Explanation for why data not available: Data are available upon reasonable request to the authors. Data use is governed by the Birhan Data Access Committee (DAC) and follows Birhan's data sharing policy. All researchers who wish to access Birhan data can complete a Birhan data request form and submit it for decision by the Birhan DAC. Datasets will only be provided with deidentified data to maintain confidentiality of study participants.
